# Brain Remodelling following Endothelin-1 Induced Stroke in Conscious Rats

**DOI:** 10.1371/journal.pone.0097007

**Published:** 2014-05-08

**Authors:** Hima C. S. Abeysinghe, Laita Bokhari, Gregory J. Dusting, Carli L. Roulston

**Affiliations:** 1 Neurotrauma Research team, Department of Medicine, St Vincent’s Campus, University of Melbourne, Victoria, Australia; 2 Department of Surgery, St Vincent’s Campus, University of Melbourne, Victoria, Australia; 3 Department of Biochemistry and Molecular Biology, Bio21 Molecular Science and Biotechnology Institute, University of Melbourne, Victoria, Australia; 4 Cytoprotection Pharmacology Program, Centre for Eye Research, The Royal Eye and Ear Hospital Melbourne, Victoria, Australia; 5 Department of Opthamology, Faculty of Medicine, University of Melbourne, Victoria, Australia; National University of Singapore, Singapore

## Abstract

The extent of stroke damage in patients affects the range of subsequent pathophysiological responses that influence recovery. Here we investigate the effect of lesion size on development of new blood vessels as well as inflammation and scar formation and cellular responses within the subventricular zone (SVZ) following transient focal ischemia in rats (*n* = 34). Endothelin-1-induced stroke resulted in neurological deficits detected between 1 and 7 days (*P*<0.001), but significant recovery was observed beyond this time. MCID image analysis revealed varying degrees of damage in the ipsilateral cortex and striatum with infarct volumes ranging from 0.76–77 mm^3^ after 14 days, where larger infarct volumes correlated with greater functional deficits up to 7 days (r = 0.53, *P*<0.05). Point counting of blood vessels within consistent sample regions revealed that increased vessel numbers correlated significantly with larger infarct volumes 14 days post-stroke in the core cortical infarct (r = 0.81, *P*<0.0001), core striatal infarct (r = 0.91, *P*<0.005) and surrounding border zones (r = 0.66, *P*<0.005; and r = 0.73, *P*<0.05). Cell proliferation within the SVZ also increased with infarct size (*P*<0.01) with a greater number of Nestin/GFAP positive cells observed extending towards the border zone in rats with larger infarcts. Lesion size correlated with both increased microglia and astrocyte activation, with severely diffuse astrocyte transition, the formation of the glial scar being more pronounced in rats with larger infarcts. Thus stroke severity affects cell proliferation within the SVZ in response to injury, which may ultimately make a further contribution to glial scar formation, an important factor to consider when developing treatment strategies that promote neurogenesis.

## Introduction

The degree of brain injury varies between each stroke victim and infarct volume has been shown to directly correlate with functional improvements 90 days post-stroke [1]. A number of events including excitotoxicity, oxidative stress, inflammation and apoptosis are initiated during and following stroke that contribute to the evolution of brain injury beyond the initial insult (*see for reviews:* [2], [3]). Evolution of infarcts tends to differ according to the underlying vascular lesion and cerebral territories involved [4]. Non-malignant infarcts evolve approximately over 3–7 days post-stroke in rodents and become stable thereafter [5], while in humans infarct evolution takes days to weeks [6], [7] with most stabilising between 1–3 months from stroke onset [8].

The importance of correctly diagnosing initial stroke severity and progression of injury relates to correct assignment of treatment options in stroke management since the initial size of a stroke lesion affects subsequent pathophysiological responses [9], [10]. To this end, prognostic clinical approaches such as the use of the NIHSS (National Institutes of Health Stroke Scale) allow prediction of functional outcome and survival in stroke patients in order to support clinical management and to correctly stratify treatment groups in clinical trials aimed at achieving neuroprotection [11]–[13]. However with the development of treatment strategies that promote brain repair, a similar comprehensive evaluation of patients following stroke will be required to assist in defining patients more likely to respond to brain restoration treatments and rule out those that lack the biological factors required to achieve improved functional outcomes [14], [15]. This highlights the need to further understand endogenous repair mechanisms and their response to different grades of stroke severity and subsequent damage.

Ischemic insults have now been shown to trigger neural progenitor cell proliferation and stem cell migration from the subventricular zone (SVZ) of the lateral ventricle to damaged regions of the brain [16], [17] even in patients of advanced age [18]. Additionally, angiogenesis is one of the pivotal restorative mechanisms initiated after ischemic injury and involves the formation of new blood vessels from the damaged vascular networks [19], [20]. Neurogenesis and angiogenesis are tightly coupled to one another and may influence brain remodelling and subsequent functional recovery in many patients following stroke [21]–[24]. The potential affect the size of the infarct has on the degree of angiogenesis has not been previously reported despite many studies focusing on the pathways involved in the induction of angiogenesis after ischemic brain injury. Likewise factors that influence the rate of neurogenesis remain to be defined. Here we investigate the effect of stroke severity on angiogenesis and SVZ cell proliferation and migration after cerebral ischemia using the endothelin-1 (ET-1) rat model of stroke, and correlate these findings with infarct volume, inflammation, scar formation and functional recovery.

## Materials and Methods

### Ethics Statement

All experiments were performed in strict accordance with the guidelines of the National Health & Medical Research Council of Australia Code of Practice for the Care and use of Animals for Experimental Purposes in Australia. The protocol was approved by the St Vincent’s Hospital animal ethics committee (AEC009/09). All surgery was performed under general anaesthesia, and paracetamol (2 mg/kg in drinking water) was provided for 24 hr prior to and after surgery in order to minimize suffering and included monitoring each rat throughout the length of the study to ensure their wellbeing.

### Surgical Preparation

A total of 37 adult male Hooded Wistar rats weighing 300–360 g (Laboratory Animal Services, University of Adelaide, Australia) were included in this study. Rats were group-housed (4 rats to a cage), until endothelin-1 induced middle cerebral artery constriction, whereupon they were housed in separate cages under diurnal lighting with ambient temperature maintained between 20 and 22°C. Rats were given free access to food and water. Rats were anaesthetised with a mixture of Ketamine/Xylazine (75 mg/kg: 10 mg/kg respectively i.p.) and maintained throughout surgery by inhalation of isoflurane (95% oxygen and 5% isoflurane). A 23-gauge stainless steel guide cannula was stereotaxically implanted into the cortex 2 mm dorsal to the right middle cerebral artery (MCA) (0.2 mm anterior, −5.9 mm lateral, −5.2 mm ventral) as in previous studies [5], [14]. Rats were allowed to recover for 5 days prior to stroke induction.

### Stroke Induction

Focal cerebral ischemia was induced in conscious rats by constriction of the right middle cerebral artery with perivascular administration of endothelin-1 (ET-1; American Peptide Company, Inc. CA, USA; 40 ρmol in 2 µl of saline over 10 min) (*n* = 34) similar to previous studies [14]. Characteristic behavioural changes indicating stroke included clenching and dragging of the contralateral forepaw and continuous circling in an anti-clockwise direction. These behavioural changes observed during stroke induction were scored 1 to 5 for stroke severity (according to our previously established protocol) with 5 being the most severe [14]. Changes in behaviour occurred routinely over 2 to 60 minutes from initiation of ET-1 infusion and rats that did not display any behavioural change were considered not to have suffered a stroke and were excluded from further study. Sham-operated rats (*n* = 3) underwent cannula implantation without ET-1 delivery.

### Assessment of Functional Outcome

Neurological assessments were conducted on all rats prior to any surgical procedures (pre-surgery), immediately prior to ET-1-induced stroke (pre-stroke) and 24, 48, 72 hrs, 7 and 14 days post-stroke. Animals were tested and scored for neurological abnormalities based on abnormal posture and hemiplegia as previously described [25], [26]. Forelimb asymmetry between the contralateral and ipsilateral forepaws was evaluated using the sticky label test by measuring latency to touch and remove attached stimuli [27]. Neurological scores from each rat post-stroke were compared to pre-stroke scores, thus each rat acted as its own control.

### Quantification of Ischemic Damage

Rats were decapitated 7 (*n* = 15) or 14 days (*n* = 19) after stroke, forebrains removed, frozen over liquid nitrogen, and stored at −80°C prior to processing. Coronal sections (16 µm thick) were prepared using a Leica cryostat (Leica Microsystems, Wetzlar, Germany) at eight pre-determined coronal planes throughout the brain from −3.2 to 6.8 mm relative to Bregma. The extent of brain injury following stroke was determined in triplicate unstained slide mounted sections using a micro computer imaging device (MCID M4 image analyser, Imaging Research Inc., St. Catharines, ON, Canada) according to the methods of Callaway and colleagues [28]. Total infarct volume was determined by integrating the cross-sectional area of damage at each stereotaxic level with the distances between levels [29]. The influence of oedema was corrected for by applying the following formula: (area of normal hemisphere/area of infarcted hemisphere) **×** area of infarct [30].

### Immunohistochemistry

Immunohistochemical staining was performed in adjacent sections to those used to quantify damage in order to identify blood vessels, proliferating SVZ cells, migrating SVZ neuroblasts, radial glial cells, as well as inflammatory cells after stroke. Control experiments included either omission of each primary antibody from the protocol or the inclusion of the appropriate IgG control to verify the specificity of each antibody. All sections were analysed with an ABC detection kit (Elite Vectastain, Vector Labs, Burlingame, CA, USA) for 30 mins followed by washes in phosphate buffered saline (PBS 0.1 M pH 7.4; 3×5 min) and 5 min incubation with diaminobenzidine (DAB; Sigma, St. Louis, MO, USA). The resultant colour reaction was visualised with an Olympus microscope (Albertslund, Denmark).

#### Blood vessel detection

For detection of blood vessels, sections were first fixed with methanol (−20°C for 15 minutes) and then washed in PBS (0.1 M; pH 7.4) containing 0.05% Tween 20 detergent (PBT) (3×5 min) followed by a 30 min block in 10% normal goat serum in PBT prior to washes in PBS (0.1 M; 3×5 min). Sections were then incubated for 1 hour with rabbit anti-von Willebrand factor (vWF; 1∶200, Millipore, Billerica, MA, USA) in PBT containing 5% NGS (0.1 M, pH 7.4) prior to washes in PBS (0.1 M; 3×5 min) and 30 min incubation with secondary antibody biotinylated goat anti-rabbit (1∶200, Vector Labs, Burlingame, CA, USA). Sections were further analysed as described above.

#### SVZ cell proliferation

For the detection of proliferating cells within the SVZ sections were fixed in 4% paraformaldehyde (PFA in 0.1 M PBS, pH 7.4, for 15 min RT) and then washed in PBS (0.1 M; 3×5 min) prior to incubation in blocking solution containing 5% NGS and 0.3% Triton X-100. Sections were then incubated with rabbit anti-Ki67 (Ki67; 1∶1000, Labvision Thermo Scientific, Fremont, CA, USA) for 1 hour in a mixture of PBS (0.1 M; pH 7.4) containing 5% NGS prior to PBS washes (0.1 M; 3×5 min) and incubation with secondary antibody biotinylated goat anti-rabbit (1∶200, Vector Labs, Burlingame, CA, USA) for 30 min. Sections were further analysed as described above.

#### Immature migrating neurons within the SVZ

For the detection of migrating immature neurons or neuroblasts within the SVZ sections were fixed in 4% paraformaldehyde (PFA in 0.1 M PBS, pH 7.4, for 15 min RT) and then washed in PBS (0.1 M; 3×5 min) prior to antigen retrieval with 10% proteinase K (DAKO, Glostrup, Denmark) in 0.1 M PBS for 3 min following by incubation in 30% fetal calf serum to stop proteinase K activity for 30 min. Sections were washed in PBS (0.1 M; 3×5 min) followed by incubation in blocking solution containing 5% NGS in 0.1 M PBS for 30 min. Sections were then incubated with guinea-pig anti-doublecortin (DCX; 1∶3000, Millipore, Billerica, MA, USA) for 1 hour in a mixture of PBS (0.1 M; pH 7.4) containing 5% NGS prior to PBS washes (0.1 M; 3×5 min) and incubated with secondary antibody biotinylated goat anti-guinea-pig (1∶200, Vector Labs, Burlingame, CA, USA) for 30 min. Sections were further analysed as described above.

#### Microglia/macrophages

For detection of microglia/macrophages, sections were fixed by serial acetone dilutions (50∶100×2 min each at RT) and washed in PBS (0.1 M; 3×5 min) prior to 30 min incubation in DAKO serum-free protein block (DAKO, Glostrup, Denmark). Sections were then incubated for 1 hour with mouse anti-OX42/CD11b (OX42; 1∶100, Serotec, Raleigh, NC, USA) prior to washes in PBS (0.1 M; 3×5 min) and 30 min incubation with secondary biotinylated goat anti-mouse (1∶200, DAKO, Glostrup, Denmark). Sections were further analysed as described above.

#### Astrocytes

For detection of astrocytes sections were fixed in 4% paraformaldehyde (PFA in 0.1 M PBS, pH 7.4, for 15 min RT) and washed in PBS (0.1 M; 3×5 min) prior to incubation in block solution containing 5% NGS and 0.3% Triton X-100 in PBS (0.1 M). Tissue sections were then incubated with mouse anti-glial fibrillary acidic protein (GFAP; 1∶400, Millipore, Billerica, MA, USA) before washing in PBS (0.1 M; 3×5 min) and incubating for 30 min with secondary antibody biotinylated goat anti-mouse (1∶200, DAKO, Glostrup, Denmark) and analysed as above.

### Immunofluorescence

Adjacent sections to those used for immunohistochemical staining, were fixed in 4% PFA for 10 min and washed in PBS (0.1 M, pH 7.4, 3×5 min). Sections were then subjected to block with DAKO serum-free protein block (Glostrup, Denmark) for 30 min. Dual immunofluorescent techniques were employed where the primary antibody for vascular endothelial cells (vWF, 1∶200) was incubated overnight at 4°C in a mixture of PBS (0.1 M, pH 7.4) containing 2% NDS, 2% NGS, 0.3% Triton X-100, together with either: mouse anti-OX42/CD11b (1∶100) to detect microglia/macrophages; mouse anti-neuron specific nuclear protein (NeuN; 1∶400, Millipore, Billerica, MA, USA) to identify neurons; or mouse anti-GFAP (1∶400, Millipore, Billerica, MA, USA) to detect astrocytes.

For characterisation of cells within the SVZ, rabbit anti-Ki67 (Ki67; 1∶2000, Labvision Thermo Scientific, Fremont, CA, USA) was co-incubated with either mouse anti-GFAP (1∶400, Millipore, Billerica, MA, USA) or guinea-pig anti-doublecortin (DCX; 1∶500, Millipore, Billerica, MA, USA); and mouse anti-Nestin (1∶400, Cell Signalling Technology, Danvers, MA, USA) was co-incubated with and rabbit anti-GFAP (1∶400, DAKO, Glostrup, Denmark) overnight at 4°C in a mixture of PBS (0.1 M, pH 7.4) containing 2% NGS and 0.3% Triton X-100.

Tissue sections were then washed in PBS (0.1 M, pH 7.4, 3×5 min) and transferred for incubation with an appropriate secondary antibody (1∶500 for all) for 2.5 hr at room temperature; Alexa-568 goat anti-rabbit and Alexa-488 donkey anti-mouse (Invitrogen Life Technologies, Grand Island, NY, USA) for the detection of vWF with either OX42 or GFAP; or Alexa-568 goat anti-mouse and Alexa-488 donkey anti-rabbit for the detection of vWF with NeuN; or Alexa-568 goat anti-rabbit and Alexa-488 goat anti-mouse for detection of GFAP with either Nestin or Ki67; or Alexa-568 goat anti-rabbit and Alexa-488 goat anti-guinea pig for detection of Ki67 with DCX. Following incubation with secondary antibodies tissue sections were washed in PBS (0.1 M, pH 7.4, 3×5 min) and then cover-slipped with DAKO fluorescent mounting medium. In control experiments, primary antibodies were omitted or the appropriate IgG control was included to verify the specificity of each antibody. Resulting sections were examined with a fluorescence microscope equipped with a 578–603 nm filter set for detection of red fluorescence and 495–519 nm filter set for the detection of green fluorescence (ZeissAxioskop2, North Ryde, Australia).

### Quantification of Immunohistochemistry

**Figure 1:**
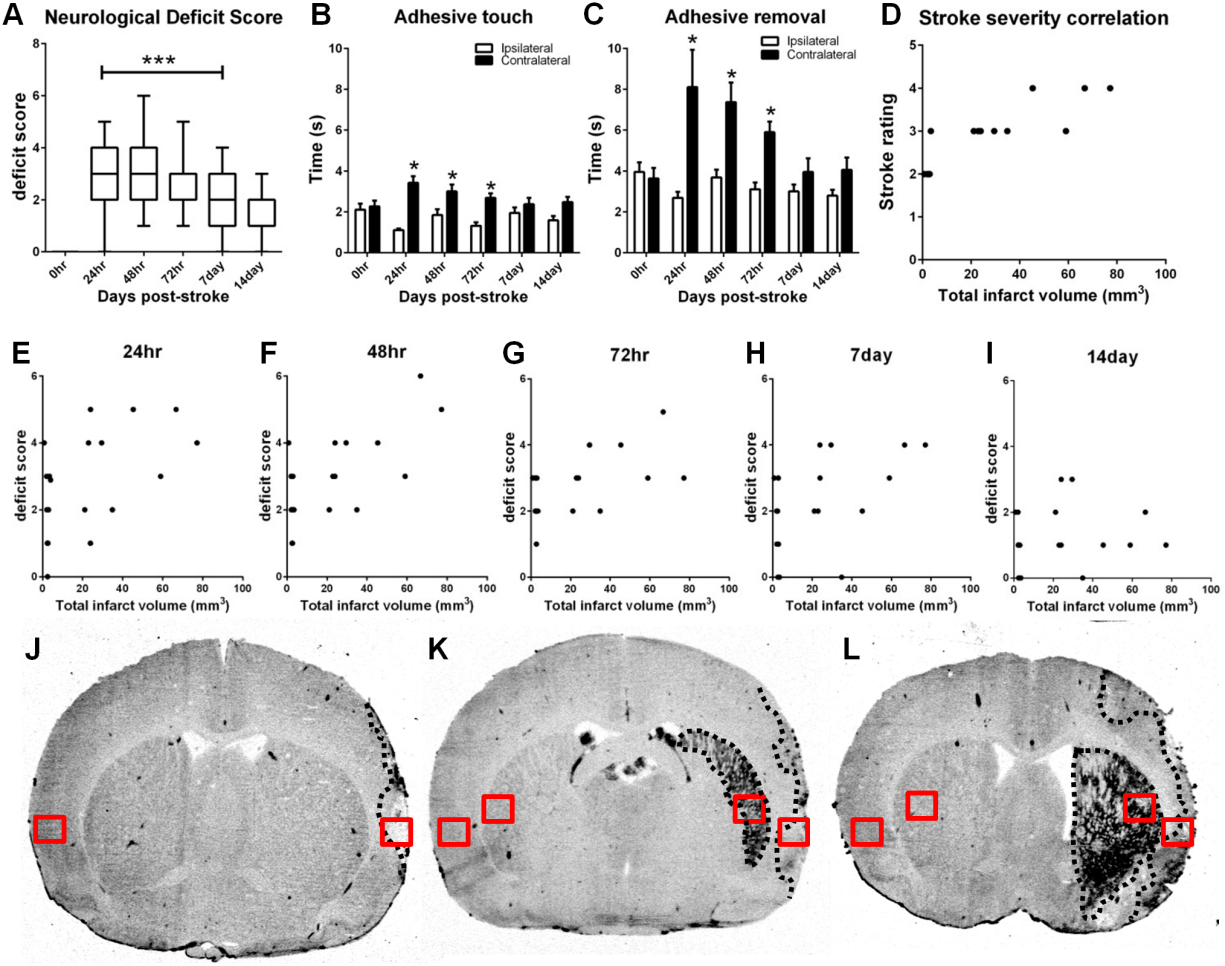
Neurological outcome 14 days after ET-1 induced stroke. Combined neurological deficit scores (A). Data presented as box plots include hinges extending from the 25^th^ to 75^th^ percentiles, the median line within the box and whiskers extending to the minimum and maximum values of the dataset. ***P<0.0001 vs 0 hr pre-stroke score (*n* = 19, non-parametric ANOVA). Latency to touch (B) and remove an adhesive (C) on the contralateral (stroke affected) forelimb compared with the ipsilateral forelimb. Data presented as mean ± SEM, *P<0.05 compared with the ipsilateral forelimb at the same time measurement (ANOVA). Scatter plots depicting correlation between infarct volumes and stroke severity rating (r = 0.88, *P*<0.0001; D) (Pearson product moment correlation coefficients). A significant correlation was found between total infarct volume and neurological deficit score at 24 (r = 0.52, *P*<0.05; E), 48 (r = 0.70, *P*<0.001; F), 72 hr (r = 0.60, *P*<0.01; G), and 7 day (r = 0.54, *P*<0.05; H) post-stroke but not thereafter (r = 0.16; I) (Pearson product moment correlation coefficients). MCID images of coronal sections taken from three animals with stroke ratings ranging from low to high; stroke rating #2 (J), #3 (K), and #4 (L) displaying damage to varying degrees within the cortex and striatum marked by the black dotted line. Consistent regions used for quantification within the infarcted cortex and striatum can be visualised by the red boxes (J–L).

Resulting tissue sections were viewed using an Olympus BH-2 microscope (Albertslund, Denmark) under x20 magnification with at least three sections per region used for assessment. MCID generated images that were used to assess infarct were used as a reference and four common regions of interest where damage was routinely observed were identified in each section for assessment: the damaged cortex, damaged striatum, and the border region surrounding the damage for both the striatum and cortex. Each area used for analysis was then compared with the appropriate corresponding mirror region in the contralateral hemisphere, as well as to sham-operated controls, using exactly the same sample area (*see* figure 1J–L).

#### Blood vessels

Blood vessel quantification was assessed 14 days post-stroke in vWF-labelled sections at two anatomical levels (+0.2 mm and −0.84 mm relative to bregma) shown in previous studies to routinely result in damage. Using digital video imaging (TK C 1480E; JVC, Wayne, NJ, USA), an automated systematic random sampling point-counting system was applied with a Computer Assisted Stereological Toolbox (CAST System; Olympus) [31]. Sampling commenced randomly within each defined field and the number of points that overlay either blood vessels or where appropriate immuno-labelled cells, was scored and then divided by the total number of points counted in each field. The number of blood vessels detected in the ipsilateral sample region was then compared to the contralateral region and expressed as 100% control. For quantification of blood vessels a common area of interest within the cortex and striatum across all stroke rats was identified based on the appearance of both small and large vWF labelled microvessel and compared directly with the contralateral mirror image.

#### SVZ cell proliferation

Ki67 positive cells within the SVZ were quantified at both 7 and 14 days post-stroke in triplicate consecutive sections (+1.2 mm bregma) to ensure the same location within the SVZ was analysed for all animals. Cells within the ipsilateral SVZ and contralateral SVZ were quantified using the cell counter plugin tool for National Institute of Health ImageJ software (USA). Images were obtained using an Olympus microscope (Albertslund, Denmark) under x20 magnification (the same magnification utilised with CAST counts). Due to a small counting frame within the SVZ total cell numbers were quantified using standard stereological techniques and ImageJ. The total number of proliferating cells detected in the ipsilateral SVZ was then compared to the corresponding contralateral SVZ and expressed as 100% control.

#### SVZ radial glial cell proliferation

Proliferating radial glial cells (Ki67/GFAP positive) within the ipsilateral SVZ and contralateral SVZ were quantified using the cell counter plugin for National Institute of Health ImageJ software (USA). Qualitative images of immature astrocytes (Nestin/GFAP positive) observed extending from the SVZ toward penumbral regions was obtained. Images were obtained using a laser scanning inverted confocal imaging system (Nikon Instruments Inc., Melville, NY, USA) under x40 magnification (a higher magnification was required to achieve greater image resolution for cell counts). For quantification, images of the SVZ were presented as collapsed reconstructions of 10 optical sections captured every 0.5 µm on the z-axis, at a scan speed of 0.5 frame/sec, a resolution of 1024×1024 pixels with the line acquisition mode. Due to a small counting frame within the SVZ cell numbers were quantified using standard stereological techniques and ImageJ. The total number of proliferating radial glial cells detected in the ipsilateral SVZ was then compared to the corresponding contralateral SVZ and expressed as 100% control.

#### SVZ neuronal cell migration

Migrating immature neurons or neuroblasts (DCX positive) within the ipsilateral SVZ and contralateral SVZ were quantified using the cell counter plugin for National Institute of Health ImageJ software (USA). DCX positive cells within the ipsilateral SVZ and contralateral SVZ were quantified using the cell counter plugin for National Institute of Health ImageJ software (USA). Images were obtained using an Olympus microscope (Albertslund, Denmark) under x20 magnification (the same magnification utilised with CAST counts). Qualitative images of proliferating immature neurons (Ki67/DCX positive) observed within the SVZ were obtained using a laser scanning inverted confocal imaging system (Nikon Instruments Inc., Melville, NY, USA) under x40 magnification (a higher magnification was required to achieve greater image resolution for cell counts). Images were presented as collapsed reconstructions of 17 optical sections captured every 0.4 µm on the z-axis, at a scan speed of 0.5 frame/sec, a resolution of 1024×1024 pixels with the line acquisition mode. Due to a small counting frame within the SVZ cell numbers were quantified using standard stereological techniques and ImageJ. The total number of immature neurons in the ipsilateral SVZ was then compared to the corresponding contralateral SVZ and expressed as 100% control.

#### Activated microglia/macrophages

Quantification of inflammatory cells was assessed within the same brain regions of interest as assessed for blood vessel quantification using the automated systematic random sampling point-counting system. The number of OX42 positive cells that displayed an amoeboid morphology with reduced processes (indicative of activation) were counted and expressed as a percentage of the total number of counts (cross hairs) within the defined sample area within the ipsilateral or contralateral hemispheres. Resting microglia displaying morphologies of small cell bodies with highly ramified processes were excluded from counts.

#### Astrocytes

GFAP labelled reactive astrogliosis was assessed and quantified based on morphological stage of activation [32]. Different gradations of reactive astrogliosis and glial scar formation were identified based on cellular hypertrophy, and length and thickness of processes and classified as either activated astrocytes or diffuse astrocytes. Quantification of activated or diffuse astrocytes within the cortical and striatal core damage and border regions was also counted using the automated systematic random sampling point-counting system and expressed as a percentage of the total number of counts (cross hairs) within the defined sample area.

### Statistical Analyses

All statistical analysis was conducted in consultation with a statistical consultant, Rachel Sore of the Statistical Consulting Centre with the University of Melbourne, Victoria, Australia. Neurological outcome data was analysed by Kruskal-Wallis non-parametric ANOVA followed by Dunn’s post-test. Hemineglect data were analysed by repeated measures for two-way ANOVA (side×hr/days after stroke) to compare latencies in the ipsilateral and contralateral forepaws over time. Individual comparisons were made using Bonferroni multiple comparisons test for all analyses where ANOVA yielded a significant result or Mann Whitney test for comparisons between two data-sets. Values are presented as mean ± SEM unless stated otherwise. To test for correlation between infarct volume and either: angiogenesis, SVZ cell proliferation, migrating SVZ neuroblasts or inflammatory cell activation, the Pearson product-moment coefficient, r, for ordinal values was determined using GraphPad Prism, version 6 (GraphPad Software Inc., San Diego CA). *P*-values less than 0.05 were considered significant.

## Results

### Functional Outcome

Significant neurological deficits were observed 24 hrs to 7 days post-stroke when compared to pre-stroke scores (*P*<0.0001 *n* = 19, non-parametric ANOVA; Figure 1A) but not beyond 7 days. Latency to touch and remove sticky labels from the stroke affected contralateral forepaw was also significantly increased when compared with the ipsilateral forelimb between 24 and 72 hrs after stroke (*P*<0.05, Two-way ANOVA; Figures 1B, C), but no significant deficits were detected after this time.

### Infarct Analysis

Histopathology 14 days after stroke revealed damage in the frontal, parietal and insular cortex and the striatum. Rats assigned lower stroke ratings during induction of stroke (ET-1 infusion) displayed damage within the cortex whereas those assigned larger stroke ratings demonstrated damage within both the cortex and striatum (Figure 1J–L) as in previous studies [14]. Rats with higher stroke severity scores exhibited significantly greater infarct volumes in both 7 and 14 day recovery groups (r = 0.88, *P*<0.0001, Figure 1D). A positive correlation between total infarct volume and neurological deficit score was also found 24 hr (r = 0.52, *P*<0.05; Figure 1E), 48 hr (r = 0.70, *P*<0.001; Figure 1F), 72 hr (r = 0.60, *P*<0.01; Figure 1G), and 7 days (r = 0.54, *P*<0.05; Figure 1H), but not at 14 days post-stroke (r = 0.16; Figure 1I).

### Angiogenesis and Infarct Correlation

**Figure 2:**
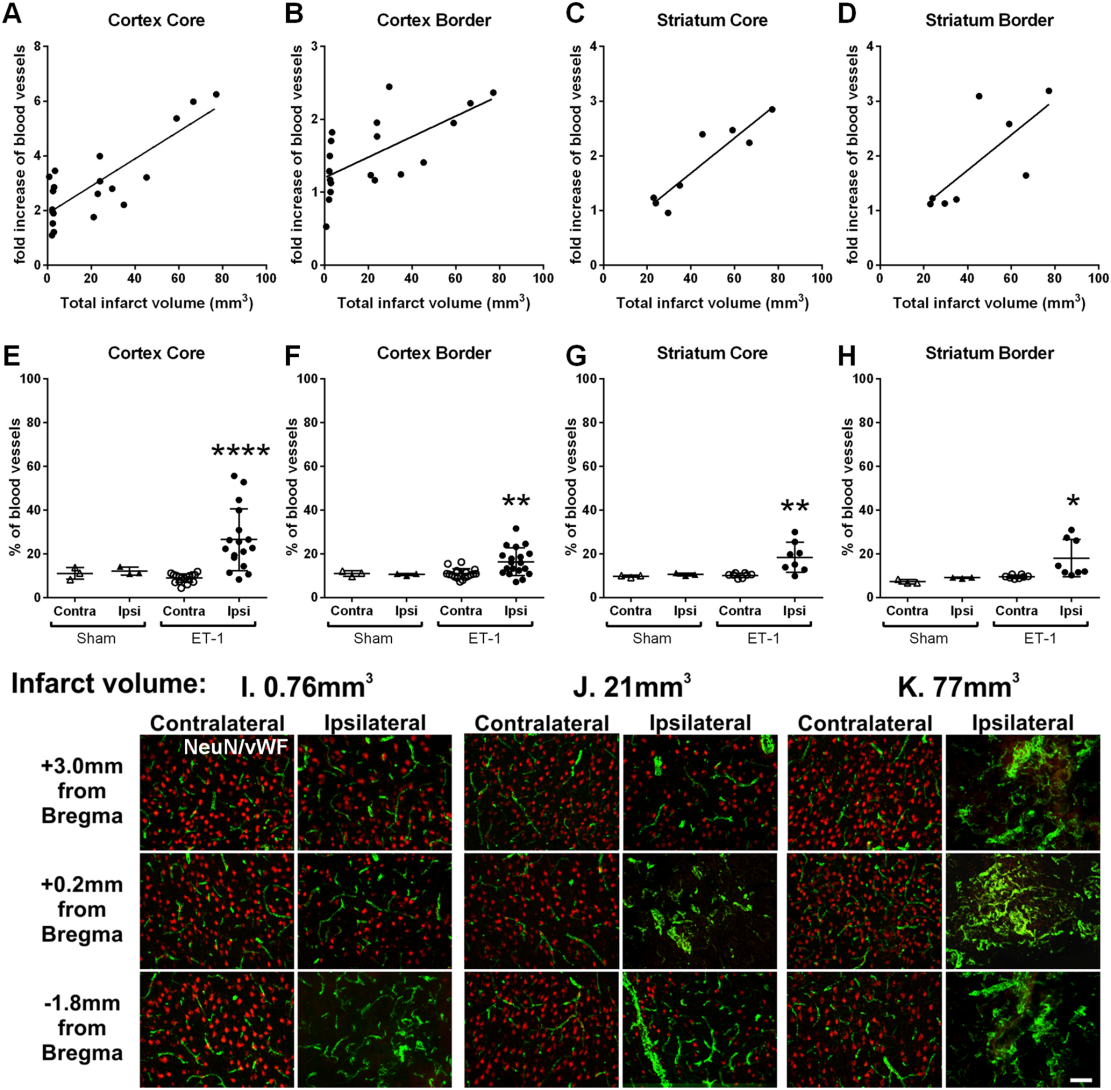
Angiogenesis and infarct volume 14 days post-stroke. Scatter plots depict a significant correlation found between degree of angiogenesis and infarct volume within cortex core (r = 0.81, *P*<0.0001, *n* = 19; A), cortex border (r = 0.66, *P*<0.005, *n* = 19; B), striatum core (r = 0.91, *P*<0.005, *n* = 8; C) and striatum border (r = 0.73, *P*<0.05, *n* = 8; D) (Pearson product moment correlation coefficients). Scatter plots of blood vessels (vWF+) from sham-operated (*n* = 3) and 14 day post-stroke animals (*n* = 19) revealed a significant increase in the number of blood vessels within the ipsilateral cortical and striatal core and border regions only (non-parametric ANOVA; E–H). Data presented as mean ± SEM, *P<0.05, **P<0.01, ****P<0.0001 compared with the contralateral hemisphere of ET-1 stroke affected animals and either hemisphere of sham-operated animals. No significant differences were observed between the contralateral hemisphere of ET-1 stroke affected animals and either hemisphere of sham-operated animals. Angiogenesis and neuronal loss within the stroke damaged brain (I–K). Immunohistochemical localisation of neurons (red) and blood vessels (green) in the cortex of stroke affected rat brains with total infarct volumes ranging from 0.76–77 mm^3^. Increased angiogenesis is observed in regions with greatest neuronal loss (ipsilateral columns I, J and K). Scale bar: I–K 100 µm.

**Figure 3:**
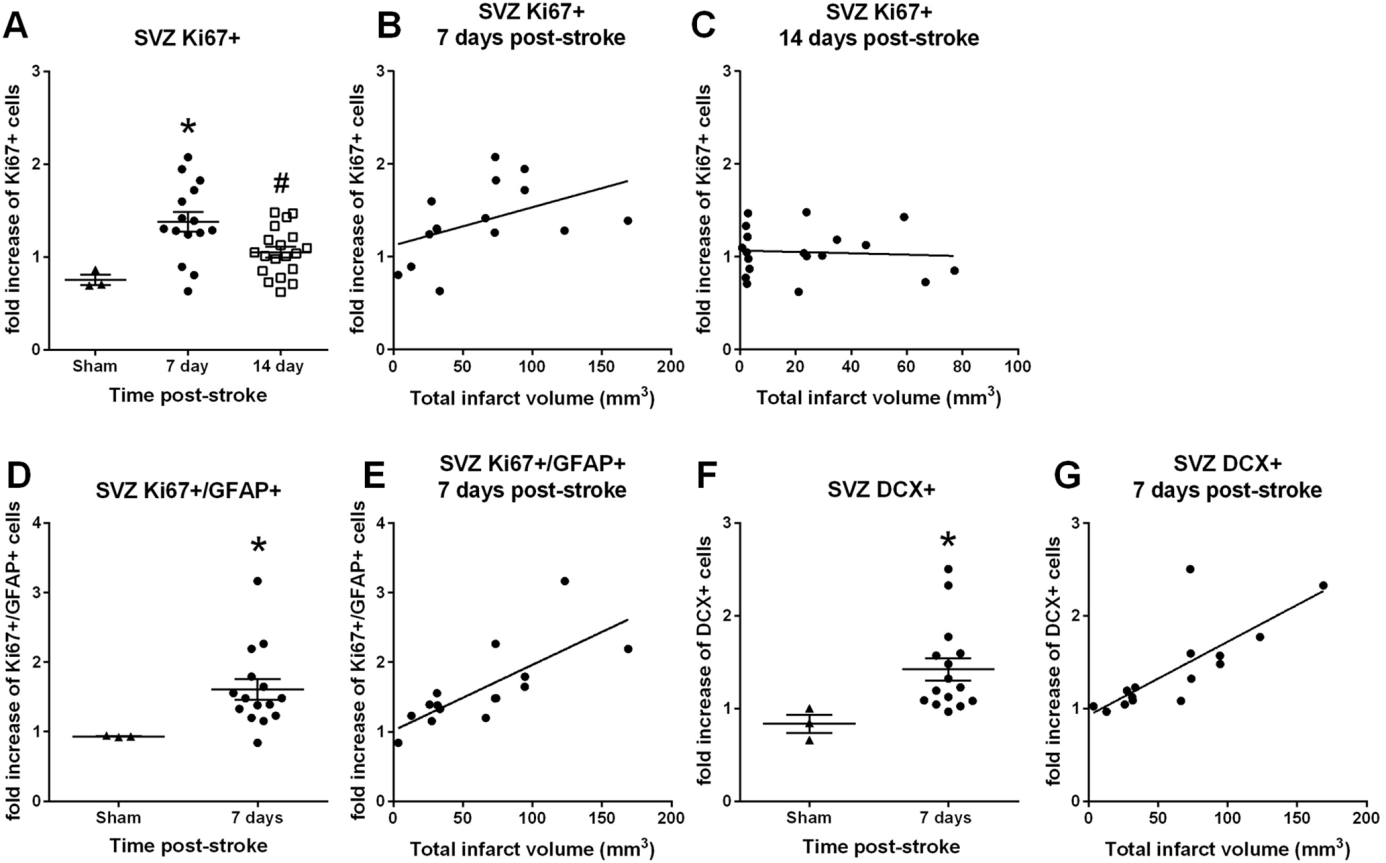
SVZ cell proliferation/migration, neural differentiation and infarct correlation 7 days post-stroke. Scatter plots of proliferating SVZ cells (Ki67+) from sham-operated (*n* = 3), 7 day post-stroke (*n* = 15) and 14 day post-stroke groups (*n* = 19) revealed a significant increase in the number of proliferating SVZ cells 7 days post-stroke (non-parametric ANOVA; A). Data presented as mean ± SEM, *P<0.05 compared with sham-operated animals and #*P*<0.05 compared with 7 day post-stroke animals. A significant correlation was found between proliferating SVZ cells (Ki67+) and infarct volume 7 days post-stroke (r = 0.73, *P*<0.005, *n = *15; B) but not at 14 days (r = 0.07; C) (Pearson product moment correlation coefficients). Scatter plots depict a significant increase in proliferating radial glial cells within the SVZ at 7 days post-stroke (Mann Whitney test; D). Data presented as mean ± SEM, *P<0.05 compared with sham-operated animals. Within the SVZ, a positive correlation was observed between infarct volumes and the number of proliferating radial glial cells (Ki67+/GFAP+; r = 0.73, *P*<0.005; E) 7 days post-stroke (Pearson product moment correlation coefficients). Scatter plots depict a significant increase in the number of immature neuronal cells within the SVZ at 7 days post-stroke (DCX+, Mann Whitney test; F). Data presented as mean ± SEM, *P<0.05 compared with sham-operated animals. Infarct volume significantly correlated with the number of immature neurons generated from the SVZ 7 days post-stroke (DCX+; r = 0.77, *P*<0.001; G) (Pearson product moment correlation coefficients).

vWF labelled blood vessels revealed regions of angiogenesis both within and around the core infarct of the cortex and where relevant in the damaged striatum. Both large and thin walled microvessels were point counted within a defined sample area (800 µm^2^) from the same anatomical region for all rats and compared to the contralateral mirror image (undamaged tissue). Blood vessel counts were then correlated with the quantified infarct volume for each rat. Positive correlations between infarct size and blood vessel numbers were detected in the ipsilateral core cortex (r = 0.81, *P*<0.0001; Figure 2A), surrounding border cortex (r = 0.66, *P*<0.005; Figure 2B), core striatum (r = 0.91, *P*<0.005; Figure 2C) and border striatum (r = 0.73, *P*<0.05; Figure 2D), with larger infarcts correlating with increased vascular density. The number of blood vessels (vWF+) significantly increased within the ipsilateral core cortex (*P*<0.0001), border cortex (*P*<0.01), core striatum (*P*<0.01) and border striatum (*P*<0.05, Scatter plots, non-parametric ANOVA; Figure 3E–H) 14 days post-stroke when compared to the corresponding contralateral hemisphere or when compared to either hemisphere of sham-operated animals. No significant differences were observed between the contralateral hemisphere of ET-1 stroke affected animals and either hemisphere of sham-operated animals (Figure 3E–H). To confirm an association between damage after stroke and angiogenesis, immunofluorescent double labelling for neurons (NeuN) and blood vessels (vWF) confirmed that greater levels of angiogenesis occurred in regions where there was greatest neuronal loss (Figure 2I–K).

### SVZ Cell Proliferation, Migration, Neural Differentiation and Infarct Correlation

In order to determine the effects of stroke on the neurogenic niche we first characterised cell proliferation within the SVZ. Ki67 positive cells were counted in both the ipsilateral and contralateral SVZ region from both 7 and 14 day recovery groups. The total number of proliferating cells within the SVZ significantly increased 7 days post-stroke compared to sham-operated animals (*P*<0.05, Scatter plots, non-parametric ANOVA; Figure 3A). This increase was no-longer detected 14 days post-stroke. At 7 days post-stroke a positive correlation was found between infarct volume and Ki67 labelled SVZ cells (r = 0.73, *P*<0.005, *n* = 15; Figure 3B), with larger infarcts associated with increased cell proliferation. No correlation between infarct volume and SVZ proliferation was found 14 days post-stroke (r = 0.07, *n* = 19; Figure 3C).

We then examined the effects of stroke on stem cell differentiation within the SVZ. We detected both Ki67/GFAP/Nestin positive radial glial cells as well as immature migrating neuronal cells identified as DCX staining. We therefore quantified Ki67/GFAP+ cells and DCX+ cells separately along the lateral ventricle in both the ipsilateral and contralateral SVZ 7 days post-stroke at the same anatomical level for all sections (+1.2 mm relative to bregma).

The number of proliferating radial glial cells (Ki67+/GFAP+) increased 7 days post-stroke compared to sham-operated animals (*P*<0.05, Scatter plots, Mann Whitney test; Figure 3D). There was also a significant correlation between infarct volume and the number of Ki67/GFAP co-labelled cells within SVZ (r = 0.73, *P*<0.005, *n* = 15; Figure 3E) 7 days post-stroke.

The number of DCX+ cells was also increased within and surrounding the SVZ 7 days post-stroke compared to sham-operated animals (*P*<0.05, Scatter plots, Mann Whitney test; Figure 3F). These DCX+ cells, did not stain with either Ki67 or GFAP and a significant correlation was also found between infarct volume and the number of DCX positive cells within the SVZ (r = 0.77, *P*<0.001, *n* = 15; Figure 3G) 7 days post-stroke.

**Figure 4:**
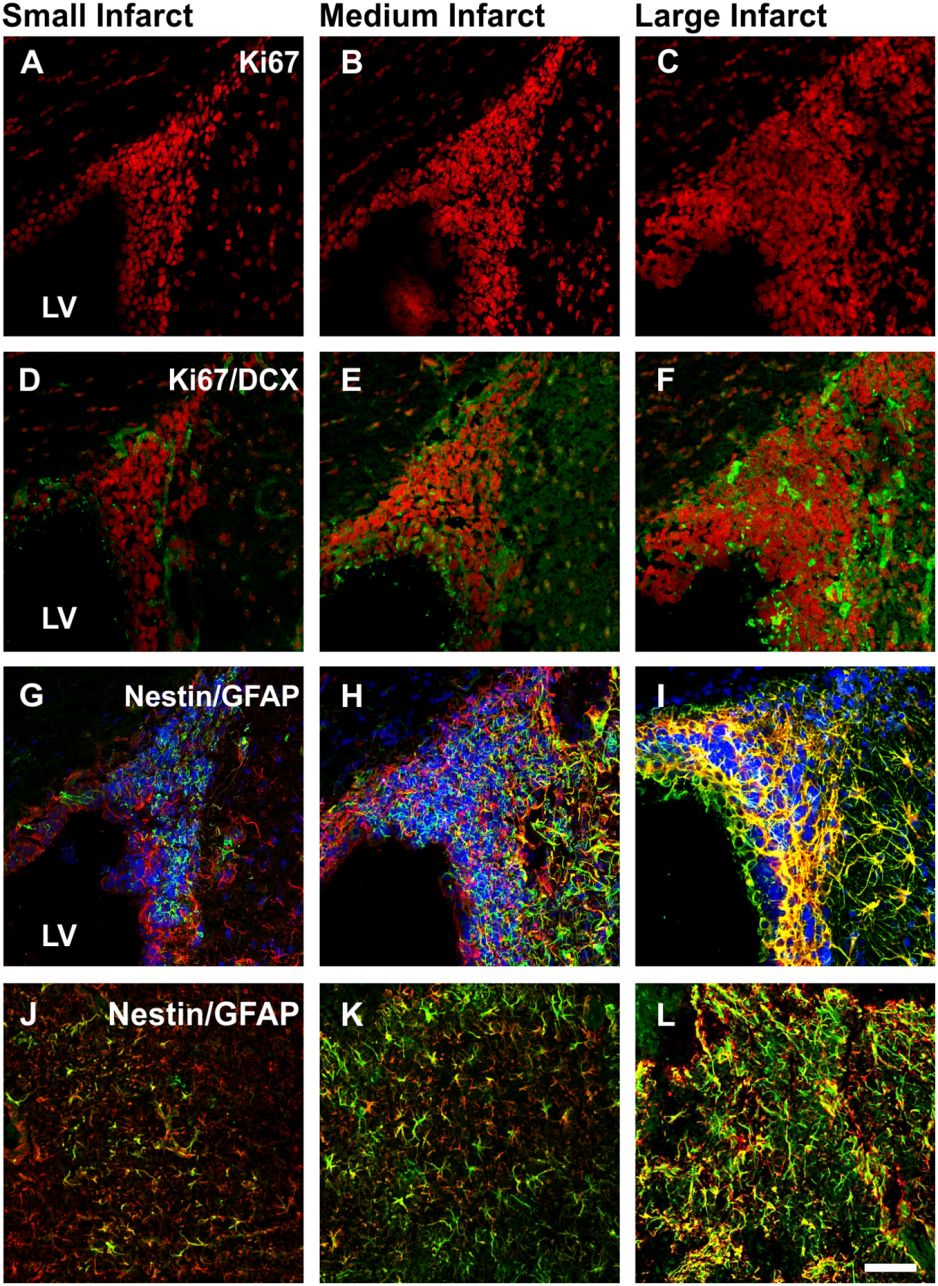
SVZ cell proliferation, migration, neural differentiation and scar formation across varied infarct volumes. Representative photomicrographs of proliferating cells (Ki67+; red; A–C) and non-proliferating immature neuronal cells (Ki67+/DCX+; red/green respectively; D–F) lining the ipsilateral SVZ, radial glial cells migrating from the ipsilateral SVZ (Nestin+/GFAP+; green/red respectively with co-expression giving a yellow appearance; G–I) and new activated astrocytes located within the cortical penumbra contributing to the glial scar (Nestin+/GFAP+; J–L). Images were taken from animals with small, medium and large infarcts. LV: Lateral ventricle. Scale bar: A–L 50 µm.

Immunofluorescence staining revealed a greater number of Ki67 positive (Figure 4A–C) and DCX positive (Figure 4D–F) cells within the SVZ of animals with larger infarct volumes. Nestin/GFAP positive immunostaining revealed a large number of radial glial cells extending from the SVZ towards the cortical and striatal penumbral regions 7 days post-stroke, with many new activated Nestin+ astrocytes detected within the border zone contributing to glial scar formation at both 7 and 14 days post stroke (Figure 4G–L). The number of Nestin/GFAP positive astrocytes detected in the border zone also appeared increased with larger infarct volumes (Figure 4J–L).

### Inflammatory Response and Infarct Correlation

#### Activated microglia/macrophages

**Figure 5:**
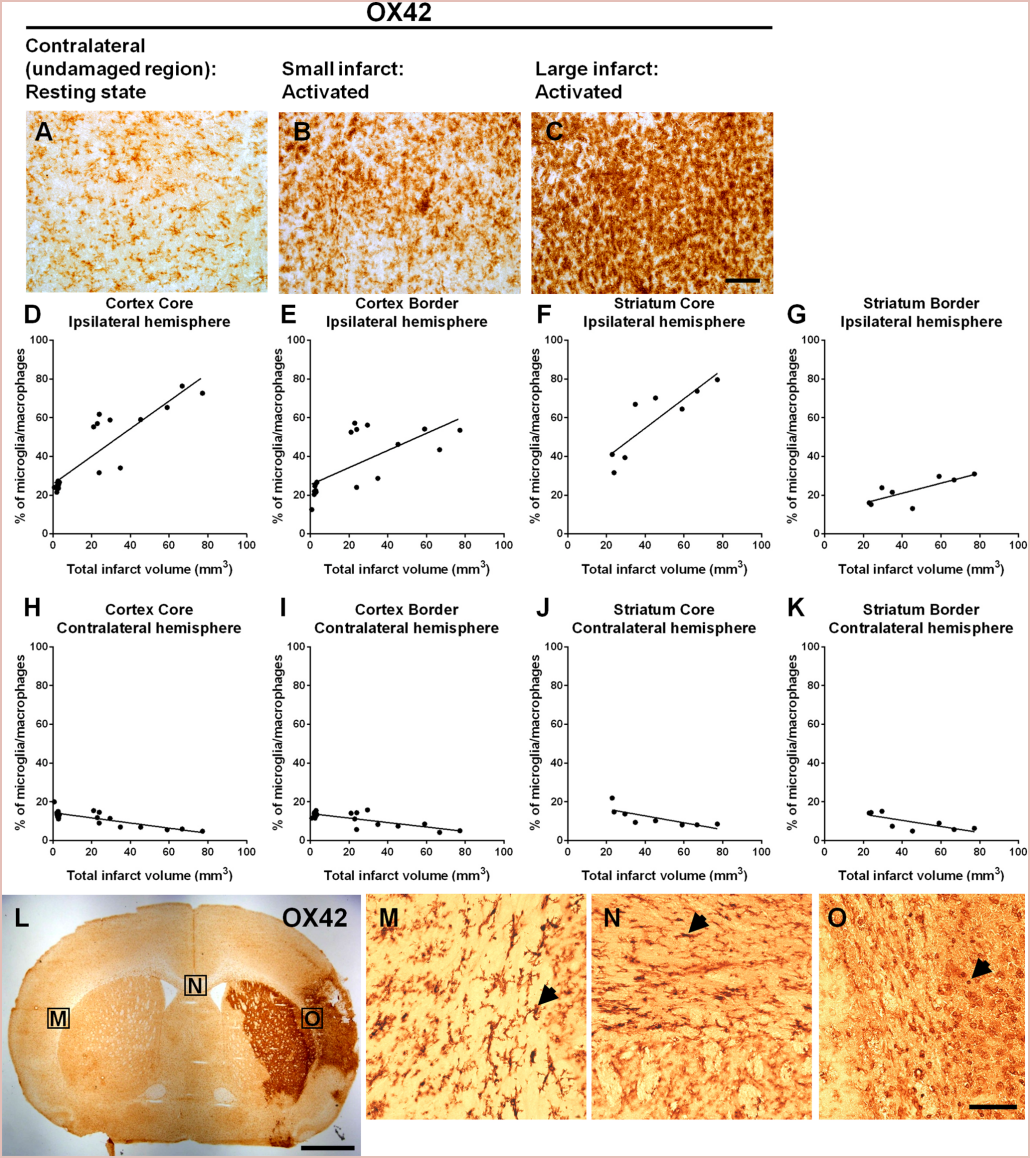
Immunohistochemical analysis of microglia and macrophages 14 days post-stroke. Immunohistochemical images of OX42 labelled microglia/macrophages within the contralateral undamaged hemisphere (A), ipsilateral core cortex of a small infarct (B) versus a large infarct (C). Within the ipsilateral hemisphere, a positive correlation was observed between infarct volume and the number of microglia and macrophages in the core cortex (r = 0.88, *P*<0.0001, *n* = 19; D), surrounding cortex (r = 0.70, *P*<0.001, *n* = 19; E), core striatum (r = 0.86, *P*<0.01, *n* = 8); F), and border striatum (r = 0.77, *P*<0.05, *n* = 8; G). In contrast, the number of microglia and macrophages in the mirror image areas on the contralateral side negatively correlated with infarct volumes within the core cortex (r = 0.81, *P*<0.0001, *n* = 19; H), border cortex (r = 0.75, *P*<0.0005, *n* = 19; I), core striatum (r = 0.75, *P*<0.05, *n* = 8; J), and border striatum (r = 0.75, *P*<0.05, *n* = 8; K) (Pearson product moment correlation coefficients). Activated microglia can be observed within the corpus callosum, and potentially represent microglia migrating from the contralateral undamaged hemisphere to sites of damage within the ipsilateral hemisphere (L). Magnified immunohistochemical images that correspond to boxes labelled (M to O) in (L) illustrate the possible migration pathway of microglia along the corpus callosum as indicated by the arrows. Scale bar: (A–C) 100 µm, (L) 2500 µm, (M–O) 50 µm.

OX42 labelling revealed both resting and activated microglia/macrophages in the contralateral and ipsilateral hemisphere respectively. Resting microglia were observed to have a ramified morphology with small cell bodies and long fine branched processes (Figure 5A) [33]. Activated microglia appeared more amoeboid in morphology with large cell bodies with retracted processes, similar in appearance to blood derived macrophages [34], [35] (Figure 5B, C). Since reactive microglia can develop into a phagocytic phenotype that is indistinguishable from infiltrating blood borne macrophages and lack discriminating cellular markers [35]–[37] both activated microglia and blood borne macrophages were quantified together. A significant increase in the number of activated microglia/macrophages was detected in the ipsilateral hemisphere up to 14 days after stroke. A positive correlation was found between the number of activated microglia/macrophages and infarct volume within the ipsilateral core cortex (r = 0.88, *P*<0.0001, *n* = 19; Figure 5D), border cortex (r = 0.70, *P*<0.001, *n* = 19; Figure 5E), core striatum (r = 0.86, *P*<0.01, *n* = 8; Figure 5F) and border striatum (r = 0.77, *P*<0.05, *n* = 8; Figure 5G) with larger infarcts containing more OX-42 positive cells within a consistent sample area. Contrastingly, within the contralateral hemisphere in regions that mirrored areas defined in the ipsilateral hemisphere, the number of activated microglia/macrophages negatively correlated with the size of infarct. This was observed within the core cortical (r = 0.81, *P*<0.0001, *n* = 19; Figure 5H), border cortex (r = 0.75, *P*<0.0005, *n* = 19; Figure 5I), core striatum (r = 0.75, *P*<0.05, *n* = 8; Figure 5J) and border striatum (r = 0.75, *P*<0.05, *n* = 8; Figure 5K). Activated microglia within the contralateral hemisphere were observed through the corpus callosum (Figure 5L–O) with increased state of activation observed closer to the site of damage.

#### Astrogliosis

**Figure 6:**
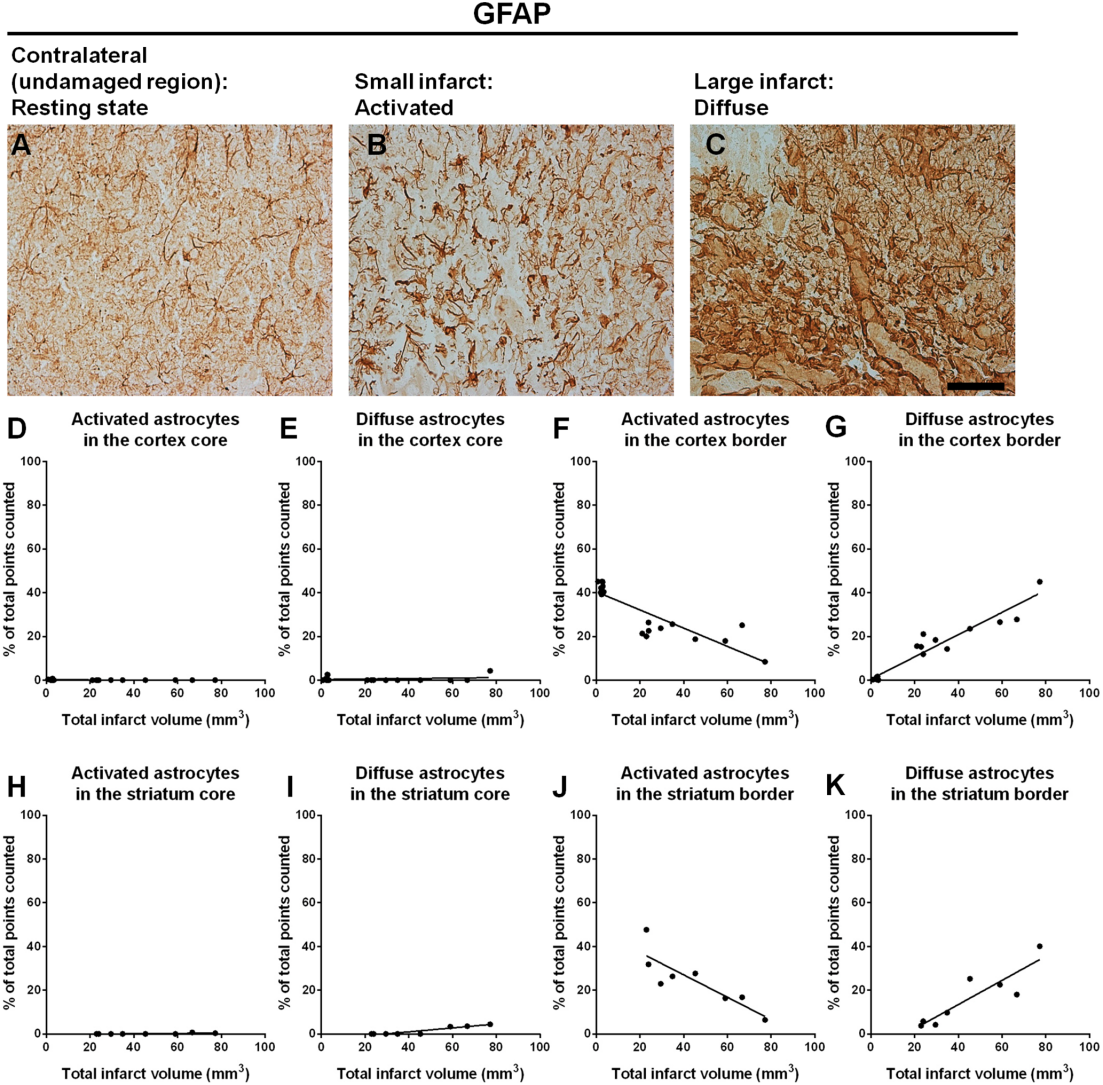
Analysis of activated and diffuse astrocytic morphologies 14 days post-stroke. Immunohistochemical images of GFAP positive astrocytes of a quiescent appearance found within the contralateral hemisphere (A), activated astrocytes surrounding a small infarct (B) and diffuse astrocytes bordering a large infarct (C). Within the core cortex, no activated astrocytes were found (D) and diffuse astrocytes were in very low numbers (E). In regions bordering the infarct, infarct volumes either negatively correlated with the number of activated astrocytes (r = 0.87, *P*<0.0001, *n* = 19; F) or positively correlated with the number of diffuse astrocytes (r = 0.96, *P*<0.0001, *n* = 19; G). No activated astrocytes were detected within the striatum core (H). A modest correlation was observed between infarct size and diffuse astrocytes within the core striatum (r = 0.93, *P*<0.001, *n* = 8; I). In areas surrounding the striatum, infarct sizes either negatively correlated with activated astrocytes (r = 0.85, *P*<0.01, *n* = 8; J) or positively correlated with diffuse astrocytes (r = 0.89, *P*<0.005, *n* = 8; K) (Pearson product moment correlation coefficients). Scale bar: A–C 100 µm.

Astrocytes were quantified based on morphological distinctions. Astrocytes of normal quiescent appearance were found in regions remote from the lesion site or within the contralateral hemisphere (Figure 6A) and were identified by low GFAP expression with long slender processes that did not overlap. Activated astrocytes were defined by an up-regulation of GFAP expression with cellular hypertrophy without pronounced overlap of processes (Figure 6B). Finally, severely diffuse astrocytes were identified based on pronounced up-regulation of GFAP expression, prominent cell body and process hypertrophy, with distinct process overlap and dense packing of cells to form a glial scar (Figure 6C). Within the core cortex, little or no activated astrocytes were observed 14 days post-stroke (Figure 6D) and diffuse astrocytes were detected in very low numbers in this region (Figure 6E). Increased GFAP immunoreactivity was predominantly observed within the border zones of the infarct. In cortical regions surrounding the infarct (border zone), infarct volumes negatively correlated with the number of moderately activated astrocytes (r = 0.87, *P*<0.0001, *n* = 19; Figure 6F) but positively correlated with diffuse astrocytes (r = 0.96, *P*<0.0001, *n* = 19; Figure 6G). Little or no activated astrocytes were observed within the core striatum 14 days post-stroke (Figure 6H). A modest correlation was observed between infarct size and diffuse astrocytes within the core striatum (r = 0.93, *P*<0.001, *n* = 8; Figure 6I). Within the border zone surrounding the striatal infarct, lesion volume negatively correlated with activated astrocytes (r = 0.85, *P*<0.01, *n* = 8; Figure 6J) but positively correlated with diffuse astrocytes (r = 0.89, *P*<0.005, *n* = 8; Figure 6K). Larger infarct volumes were associated with greater glial scar formation encircling the damaged striatum and cortex.

## Discussion

In the present study we confirm that the ET-1 model of stroke is a valuable tool for studying brain remodelling and long term recovery, specifically angiogenesis, microglia/macrophage activation, astrocytic morphological transition and SVZ cell proliferation, differentiation and migration. There is a positive correlation between lesion size and proliferation and differentiation of cells within the SVZ 7 days post-stroke, as well as between lesion size and the degree of angiogenesis, microglia activation and astrogliosis 14 days after stroke. This confirms and extends the initial report of Nagai *et al* [9] who showed pathological responses initiated by stroke affects the magnitude of the inflammatory response, and identifies the effect of stroke severity on subsequent repair mechanisms, important factors to consider when exploring treatment strategies that promote recovery of the injured brain.

### Predictive Outcome and Long Term Recovery

Neurological deficit scores after stroke have commonly been used as a predictor of long term outcomes [38], [39]. The current scales however, do not take into account spontaneous recovery of functions that occur weeks to months after stroke without change to the resulting brain infarct. None-the-less use of such scales has proven beneficial when assigning patients to treatment. We have previously reported the benefits of predicting stroke outcome using the ET-1 model of stroke in conscious rats, where variability in stroke severity reliably correlates with infarct volumes up to 3 day post-stroke [14]. To this end stroke outcomes using the ET-1 model allow clinically relevant assessments of stroke pathology since stroke in humans is highly variable: a large infarct can result following occlusion of a major artery such as the MCA or a small infarct can result due to occlusion of a single penetrating artery [40]. Predicting stroke outcome using rodent models has also been described where neurological deficits are tested upon recovery from anaesthesia [41]. In the present study we demonstrate that whilst a neurological scale at the time of stroke can be reliably used to predict histological outcome up to 14 days, it is not a reliable long-term predictor of neurological outcome, since recovery of deficits observed 14 days after stroke did not correlate with reduced lesion size.

### Angiogenesis and Infarct Size

The potential for brain regeneration relies heavily on the surrounding microenvironment and for neuronal replacement to occur it requires supporting vasculature. Angiogenesis is a fundamental process occurring during development and in wound healing in adults, and previous studies using the MCA thread occlusion model have reported new vasculature develops mostly within the peri-infarct zone [19], [42]. In contrast, we showed evidence of angiogenesis across the entire infarct in addition to the border zone [5], similar to that which occurs in humans where a significantly higher microvessel density is observed across the damaged region of the brain [20]. We now demonstrate that larger infarct volumes result in increased microvessel density within the core infarct as well as the peri-infarct zone, suggesting that even the most severely damaged brain might be capable of supporting neuronal replacement.

Previously the degree of angiogenesis, measured by increased cerebral blood flow and microvessel density, has been correlated with longer survival in stroke affected patients [20] suggesting that active angiogenesis may be beneficial to the damaged brain. Indeed we have previously reported that with spontaneous functional recovery newly formed vascular networks are detected within damaged regions [5]. One possible mechanism may be associated with the damage sustained to cerebral blood vessels themselves, such that a greater degree of vascular sprouting may result from an increased number of damaged vessels. Cerebral ischemia initially compromises blood vessels and may result in extensive damage to the vessel wall. In the present study we confirmed that a greater degree of angiogenesis occurs in regions associated with extensive neuronal loss and increased stroke severity. Thus increased vascular damage is associated with increased neuronal loss, resulting in increased angiogenesis.

The angiogenic process can improve the perfusion of oxygen and nutrients to ischemic and surrounding tissues where partially affected neurons that have the capability of surviving reside, which in turn may improve functional outcome following stroke [19]. Proliferation of endothelial cells within damaged brain regions are initiated within days after ischemic events [43], [44] and the process of active angiogenesis may be beneficial for the ischemic brain. Interestingly, the correlation between angiogenesis and improved neurological outcome following stroke has been observed in both animal stroke models and human stroke patients [20], [45], [46]. Angiogenesis has also been linked directly with other endogenous recovery mechanisms including neurogenesis, synaptogenesis and neuronal and synaptic plasticity which are all events involved in the long-term repair and restoration process of the brain after stroke. If these repair processes are not achieved then vessel density may later regress. Clearly angiogenesis is a potential target for improved regenerative recovery after ischemic stroke.

### Neurogenesis and Infarct Size

Ischemic insults to the brain have now been shown to trigger progenitor cell proliferation and stem cell migration from the SVZ of the lateral ventricle to damaged regions of the brain. We saw increased SVZ cell proliferation in the ipsilateral hemisphere 7 days post-stroke. Importantly, rats with larger infarcts were shown to have a greater degree of SVZ cell proliferation. Previous studies have shown proliferating SVZ stem cells increase as early as 2 days post-stoke, an effect that peaks by 7 days and returns to normal by 14 days [47], [48]. Ours is the first study to show that this effect is initially amplified with increasing stroke severity, but is not sustained beyond this time.

The adult SVZ contains a heterogeneous population of cells including SVZ radial glial cells (type B cells), rapidly dividing transient amplifying cells (type C cells) and migratory neuroblasts (type A cells) [49], [50]. Normally within the adult SVZ, newly generated stem cells migrate through the rostral migratory stream (RMS) into the olfactory bulb and differentiate into interneurons [51]. Cerebral ischemia alters the migration pathway and neuroblasts generated from the SVZ migrate to the injured brain regions instead, where only a few differentiate into new neurons (<0.2%) [48]. In the present study rats with larger infarcts showed evidence of increased SVZ proliferation with a greater number of cells generated following a neuronal differentiation path as evidenced by increased DCX staining. DCX positive neuroblasts were not however evident within penumbral regions (Figure S1D–G), possibly suggesting a low survival rate of these new neurons with greater stroke severity. Arvidsson *et al.* [48] previously described the unfavourable hostile environment of severely ischemic tissue for newly formed neurons.

A notable loop of interactions between newly formed stem cells and blood vessels has been reported [24] and this interaction may be magnified with larger infarct volumes. Activated endothelial cells, injured and newly forming blood vessels release an array of trophic factors including vascular endothelial growth factor (VEGF) and brain-derived neurotrophic factor (BDNF) which can evoke neurogenesis, are chemotactic for progenitor cells and may enhance the survival and integration of neuroblasts into the brain tissue [18], [22], [52].

In the present study larger infarct volumes also correlated with an increase in the number of radial glial cells generated within the SVZ 7 days post-stroke. Radial glial cells retain neural stem cell-like properties and are capable of migrating from the SVZ towards the damaged brain where they can differentiate into either neurons or astrocytes based on the microenvironment [50], [53], [54]. Our results demonstrate that an increase in the number of SVZ derived radial glial cells 7 days post-stroke appear to be extending towards cortical and striatal penumbral regions of animals with larger infarcts (Figure S1A–C). By 14 days Nestin expressing reactive astrocytes are detected within the glial scar and possibly indicate a new cell population of reactive astrocytes that have migrated from the SVZ to the site of injury in response to stroke. These results indicate that without intervention, newly generated stem cells extend towards the damaged brain but have a greater propensity to become astrocytes that in turn contribute to glial scar formation that poses a major obstacle to brain repair.

### Microglial Response to Lesion Size

Previous studies have reported a positive correlation between infarct size and microglia activation up to 7 days following discrete photochemically induced thrombotic lesions of the cerebral cortex [9]. We now show that this effect is also true for brain injury following focal ischemia using the ET-1 model with a 14 day recovery. Furthermore, we observed within the contralateral hemisphere a decrease in the percentage of activated microglia/macrophages in rats with larger infarcts. Microglia can become motile and actively move to the site of injury following chemotactic signals [55], [56] and have also been found to migrate through the corpus callosum to arrive to their desired destination [57]. Indeed in the present study we observed in rats a reduction in the number of microglia in the contralateral hemisphere as well as graded changes in morphological activation throughout the corpus collasum, suggesting movement of microglia from uninjured brain regions to the infarcted hemisphere as described by others [55], [56]. Therefore, the accumulation of microglia within the infarcted region at 14 days is likely to comprise of proliferating resident microglia as well as migrating microglia [58].

The accumulation of activated microglia/macrophages within ischemic regions occurs early after stroke with a peak response occurring at 7 days [59] when the inflammatory response peaks [5]. Our data now indicate a significant inflammatory response persists 14 days post-stroke where the size of the lesion still dictates the level of inflammatory cell activation. Persistent microglial activation can become maladaptive with the release of cytotoxic molecules [60], [61] and pro-inflammatory cytokines that have been found to be neurotoxic [2], [62], [63]. In contrast, recent reports suggest that activated microglia may also play a role in brain repair. For it has been implicated in increasing neurogenesis *in vitro* [64] and *in vivo* [65] following stroke, and free radicals derived from activated microglia might also have a role in promoting angiogenesis [5]. Microglia may also exert neuroprotection by producing neurotrophic molecules such as brain-derived neurotrophic factor (BDNF), insulin-like growth factor 1 (IGF-1), and several other growth factors [66]. However the degree of injury may be an important stimulus for microglia, determining whether they play a detrimental or beneficial role. Establishing the microglia/macrophage response after an ischemic insult will assist in the understanding of endogenous processes occurring to highlight the positive and negative influences these inflammatory cells may have on endogenous repair mechanisms that support neuronal differentiation and survival of migrating neural progenitor cells.

### Astrocytic Response to Varying Infarct Volumes

Positive correlations between lesion size and astrocytic activation has also been reported by Nagai *et al* [9]. Quantification of activated astrocytes according to morphological transition in correlation with infarct volume has not been previously reported. Astrogliosis following injury to the brain is not a simple all-or-none phenomenon, but a finely gradated continuum of morphological change that can either influence brain preservation or result in long-lasting scar formation and reorganisation of tissue structure [32], [67]. We now confirm that larger infarct volumes contain an increased percentage of severely diffuse reactive astrocytes, and a decrease in the percentage of moderately activated astrocytes within border regions of the infarct.

Astrocytes with moderately activated morphologies have been thought to initially provide trophic and protective support to neurons [68] that may promote neuroprotection and could possibly create an environment conducive to facilitate neurite extension and axonal outgrowth to aid in regeneration [69]. If immediate restoration of blood flow after initial stroke insult is initiated, astrocytes of mild or moderately active morphologies have the potential to resolve and return to their appearance in healthy tissue once the triggering mechanism is resolved [70]. On the other hand if the insult continues to persist without resolution, astrocytes transition into severely diffuse reactive distinctions and may not revert to their previous morphological states [70]. These severely diffuse reactive astrocytes may no longer be beneficial to neurons and can instead majorly impede axonal regrowth through formation of the glial scar and secretion of extracellular matrix molecules that prevent axonal regeneration [67], [71]–[73].

Based on our findings larger infarcts induce astrocytic transition to severely diffuse morphologies that could possibly contribute to the spread of damage as well as combining to form a physical barrier to brain remodelling and repair. Importantly we now show that increased cell proliferation within the SVZ of rats with larger infarcts contribute to increased glial scar formation since we observed a greater proportion of Nestin/GFAP positive cells extending from the SVZ as well as within the border zone. Much focus in developing treatment strategies that encourage brain repair has been to target increased proliferation and migration of neural progenitor cells from the SVZ after brain injury [74]–[78]. Treatments aimed towards up-regulating endogenous proliferation from either the SVZ or dentate gyrus of the hippocampus should also confirm the fate of the newly generated cells to appropriately assess the therapeutic benefit [79]. Given the results presented in the current study it is now clearly important to characterise the long term impact of these treatments across differing stroke severities. Particularly since increasing stem cell proliferation and migration may be counterproductive if the microenvironment supports conversion to astrocytes that accentuate the glial scar rather than conversion to new neurons.

## Conclusion

The potential for brain regeneration relies heavily on the surrounding microenvironment and this includes the pathogenic contribution from inflammatory cells, angiogenesis and neural stem cells. Ours is the first study to demonstrate a positive correlation between stroke severity and the effects on pathological responses to brain recovery using a focal model of cerebral ischemia. In addition, our results have highlighted the importance of taking into consideration infarct size when developing therapies that are targeted towards promoting neurogenesis. Therapeutic strategies aimed at increasing cell proliferation and migration from the SVZ need to pay particular attention to the phenotypic transformation of neural progenitor cells following treatments to avoid unwanted additional scar formation. Understanding the endogenous pathways initiated after ischemic insult will enable us to design better therapies to augment the restorative power of the cerebral vasculature as well as improve brain remodelling for improved functional outcomes.

## Supporting Information

Figure S1 **SVZ cell extension towards infarct and neural differentiation.** Photomicrographs of the contralateral (A) and ipsilateral (B) hemispheres depicting radial glial cells mainly within the ipsilateral hemisphere extending from the SVZ towards the cortical and striatal penumbral regions (Nestin+/GFAP+; green/red respectively with co-expression giving a yellow appearance). Core infarct regions are marked by a white dotted line. Merged immunofluorescent image of radial glial cells extending towards the penumbra from the SVZ as indicated by the arrows (Nestin/GFAP/DAPI; green/red/blue respectively with Nestin/GFAP co-expression giving a yellow appearance; C). Immunofluorescent images depicting immature neuronal cells (DAPI/DCX+; blue/green respectively; D–G) within the penumbra cortex (D) and SVZ of the ipsilateral hemisphere (E) and mirror images of the contralateral hemisphere (F, G). All images were taken from animals with large infarcts. LV: Lateral ventricle, Ctx: Cortex, Stm: Striatum. Scale bar: A,B 400 µm, C 40 µm, D, G 100 µm, E, F 50 µm.
(TIF)
