# Supplementary figures and images for: Brain Remodelling following Endothelin-1 Induced Stroke in Conscious Rats
Source: PLoS One. 2014 May 8;9(5):e97007. doi: 10.1371/journal.pone.0097007 (PMC4029108; doi:10.1371/journal.pone.0097007)

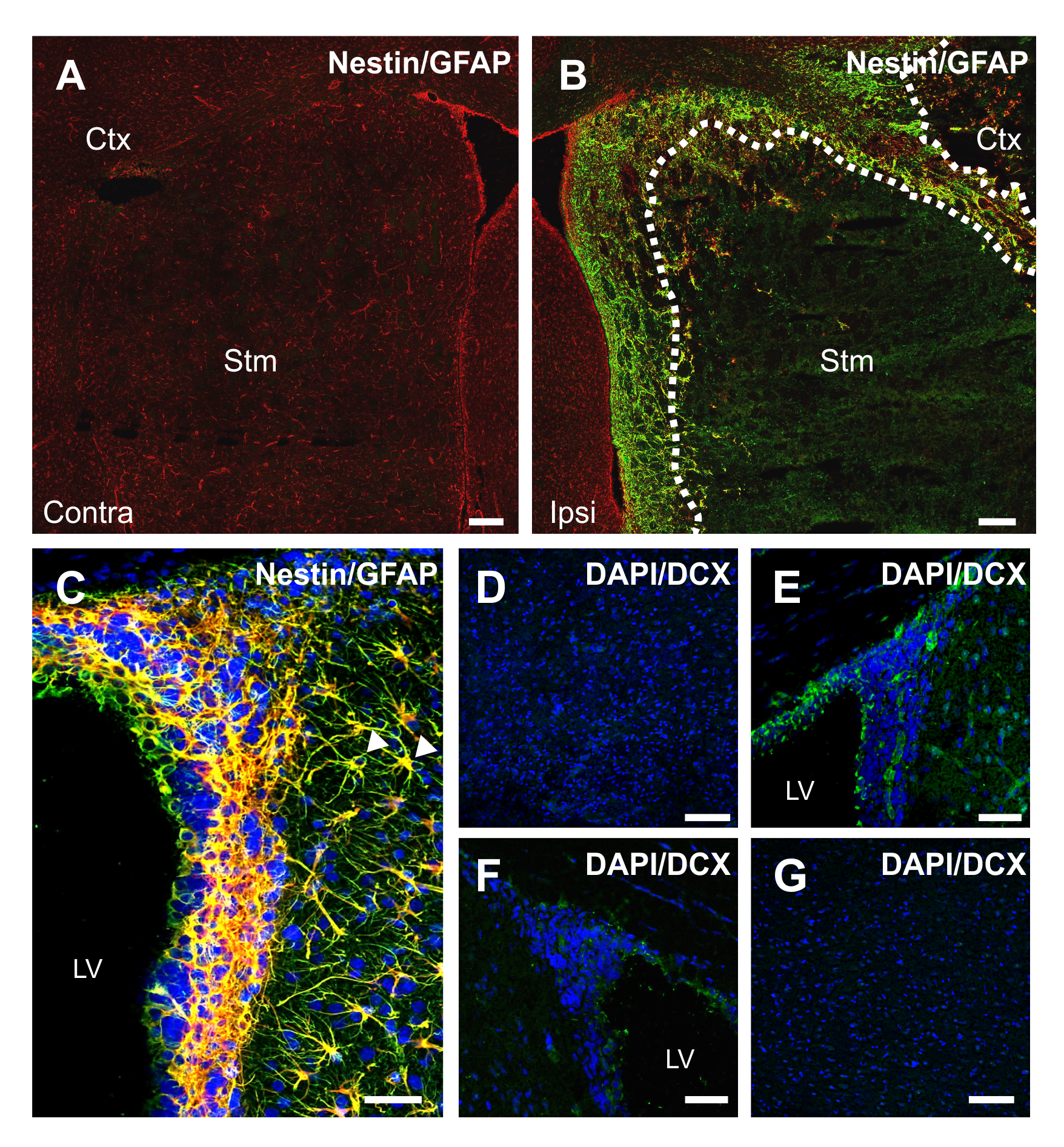

Supplement: Figure S1 — SVZ cell extension towards infarct and neural differentiation. Photomicrographs of the contralateral (A) and ipsilateral (B) hemispheres depicting radial glial cells mainly within the ipsilateral hemisphere extending from the SVZ towards the cortical and striatal penumbral regions (Nestin+/GFAP+; green/red respectively with co-expression giving a yellow appearance). Core infarct regions are marked by a white dotted line. Merged immunofluorescent image of radial glial cells extending towards the penumbra from the SVZ as indicated by the arrows (Nestin/GFAP/DAPI; green/red/blue respectively with Nestin/GFAP co-expression giving a yellow appearance; C). Immunofluorescent images depicting immature neuronal cells (DAPI/DCX+; blue/green respectively; D–G) within the penumbra cortex (D) and SVZ of the ipsilateral hemisphere (E) and mirror images of the contralateral hemisphere (F, G). All images were taken from animals with large infarcts. LV: Lateral ventricle, Ctx: Cortex, Stm: Striatum. Scale bar: A,B 400 µm, C 40 µm, D, G 100 µm, E, F 50 µm. (TIF) [file pone.0097007.s001.tif]
